# Counting general practitioners: a comparative repeat cross-sectional analysis of GPs in NHS general practice in England

**DOI:** 10.3399/BJGP.2024.0833

**Published:** 2025-10-21

**Authors:** Luisa M Pettigrew, Soraya Akl, Aamena Valiji Bharmal, Josephine Exley, Luke N Allen, Irene Petersen, David A Cromwell, Nicholas Mays

**Affiliations:** 1 Department of Health Services Research and Policy, London School of Hygiene & Tropical Medicine, London, UK; 2 The Health Foundation, London, UK; 3 School of Primary Care and Public Health, Imperial College London, London, UK; 4 Nuffield Department of Primary Care Health Sciences, University of Oxford, Oxford, UK; 5 Department of Primary Care and Population Health, University College London, London, UK; 6 Department of Clinical Epidemiology, Aarhus University, Aarhus, Denmark

**Keywords:** general practice, human resources, primary health care, workforce

## Abstract

**Background:**

There have been successive government promises to increase general practitioner (GP) numbers in England.

**Aim:**

To compare how NHS general practice GP numbers and trends differ depending on how GPs are defined and data are analysed.

**Design and setting:**

This was a comparative repeat cross-sectional study of NHS general practice GP numbers in England.

**Method:**

The study compared NHS England’s General Practice Workforce GP data quarterly between September 2015 and September 2024 by headcount and full-time equivalent (FTE); with and without general practice trainees; and relative to population size.

**Results:**

Between September 2015 and September 2024, if counting fully qualified GPs and general practice trainees, there was an 18% (41 193 to 48 758) rise in numbers; whereas if fully qualified FTE GPs alone were counted there was a 5% reduction (29 364 to 27 966). Once growth of the population registered with an NHS general practice was considered, the trend in GPs per capita varied between a 6% rise or 15% reduction. There was an increasing difference in the number of patients per GP between practices, with a 5th to 95th percentile range of 1204 and 4139 patients per fully qualified FTE GP in 2015; by 2024 these percentiles increased to 1357 and 5559. Using Office for National Statistics (ONS) mid-year population estimates produced different results as their population estimates are lower than the total number of patients registered with an NHS general practice.

**Conclusion:**

How GPs are defined, whether working hours are considered, and what measure of population size is used affects the interpretation of workforce trends. Using fully qualified FTE GPs per capita most closely reflects GP capacity, although there are limitations to current NHS data. Reporting the spread of patients per GP at practice level is necessary to capture the widening variation in GP provision in England.

## How this fits in

The numbers of GPs in NHS general practice depend on how GPs are defined and how data are analysed. This article provides a comprehensive picture of trends in GP capacity in English NHS general practice between 2015 and 2024. It shows that the number of fully qualified GPs working in NHS general practice is not keeping pace with population growth and there is increasing variation in the number of patients per GP between practices. Research and policy recommendations to improve the consistency and clarity of reporting GP workforce statistics are offered.

## Introduction

Statistics on GP numbers are essential to inform workforce planning and policy evaluation at a local and national level. Although England has relatively comprehensive NHS general practice workforce data compared with other countries, counting and reporting GPs is not straightforward.^1–3^ Statistics rely on accurate data collection and there are various options for analysis. For example, there are different definitions of a GP working in NHS general practice, whether reported working hours are considered, and whether GP figures are reported as absolute numbers or relative to the population. These differences can result in stakeholders citing figures inconsistently and at cross purposes.^4–7^

As part of their contractual requirements, NHS general practices in England are required to submit monthly workforce data via the National Workforce Reporting Service (NWRS) online portal.^8^ NHS England (NHSE) compiles submissions and publishes individual GP-level data, in a non-identifiable way, to provide total national and regional NHS general practice workforce figures. It also publishes data at practice level with the associated number of NHS-registered patients. Workforce figures are reported by headcount and full-time equivalent (FTE). FTE figures represent the proportion of hours worked out of the total number of hours considered to be full-time, which NHSE considers as 37.5 h per week for a fully qualified GP and 40 h per week for a GP trainee. It adjusts GP trainees’ FTE to be comparable with that of fully qualified GPs. The category of ‘All GPs’ reported by NHSE includes ‘GPs in training grades’ (referred to in this paper as ‘GP trainees’), Foundation Years 1 and 2 doctors, GP specialty registrars and any other ‘Junior Doctor’ working in NHS general practice. NHSE publishes details of fully qualified GPs’ role (that is, partner, salaried, regular locum, or retainer), and provides data by gender, age band, and place of primary medical qualification. *Ad hoc* short-term GP locums (for example, covering holidays) and GPs employed via primary care networks (PCNs) are reported separately by NHSE.

To illustrate the consequences of counting GPs in different ways, this study compared how English NHS general practice GP numbers and trends over time depend on how GPs are defined and workforce data are analysed. This study demonstrates how different approaches have an impact on estimates of trends over time and patterns across general practices.

## Method

The study used NHSE’s ‘General Practice Workforce’ September 2024 Bulletin Tables’ quarterly data from September 2015 to September 2024 to compare the total number of GPs in NHS general practice by headcount and FTE, with and without GP trainees and per 1000 NHS-registered patients, nationally. The study also calculated GPs in NHS general practice per 1000 patients using Office for National Statistics (ONS) mid-year population estimates between 2017 and 2023, when mid-year ONS and June workforce data were both available. Practice-level NHS workforce data from September 2015 to September 2024 were used to calculate the number of NHS-registered patients per fully qualified (that is, excluding GP trainees) FTE GP in NHS general practices.

For practice-level analysis, practices with missing data or <1000 patients were excluded as these are likely to be atypical, for example, closing or caring for an atypical population. On average, 2.5% (range 1–6%) of practices were excluded from analysis each quarter (Supplementary Table S1). During this period, the number of unique practices also fell from 7623 to 6256 because of closures or mergers.^3^ No workforce data were available for December 2015 and June 2016. Data were extracted by one author (the first author) and cross-checked by another (the second author).

Trends over time were analysed using linear regression analysis and report the absolute change per quarter coefficient with 95% confidence intervals (CIs). Practice-level NHS patients per fully qualified FTE GP in NHS general practice figures (patient-to-GP ratios) stratified between the 5th and 95th percentiles of practices are presented and the distribution in September 2015 and September 2024 compared using histograms to illustrate the change in the extent of variation across England over the 9-year period. Practice-level fully qualified FTE GP per 1000 NHS patients (GP-to-patient ratios) analyses are provided in supplementary material (Supplementary Figure 3).

Stata 18 was used for analysis. Findings are reported using the ‘REporting of studies Conducted using Observational Routinely-collected Data’ (RECORD) guidelines.^9^

## Results

### Total GPs by headcount, with and without GP trainees

**Figure 1.**
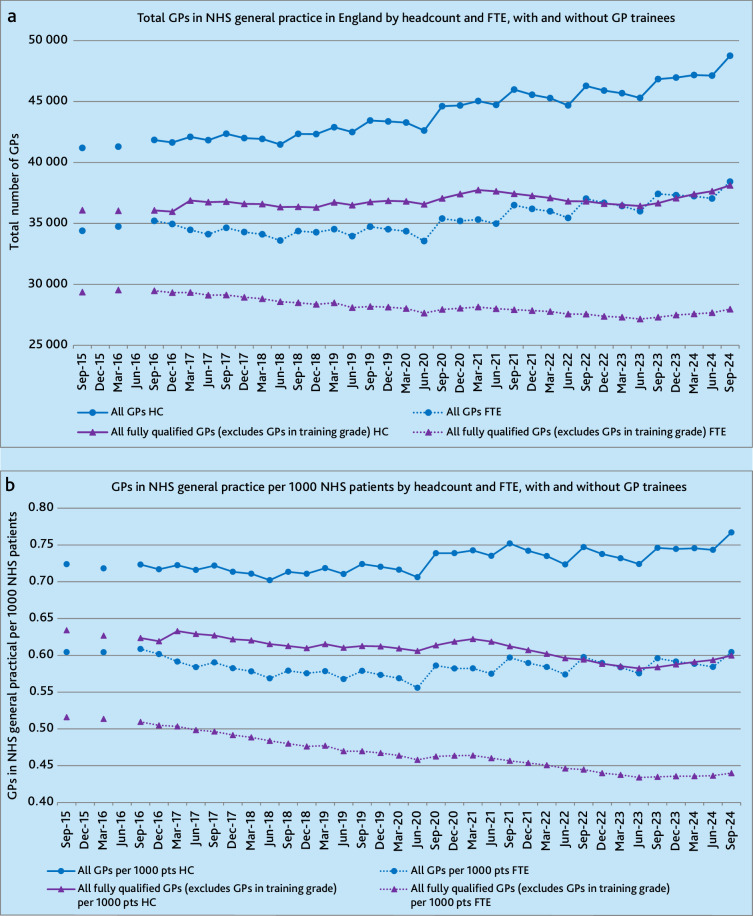
(a) Total GPs in NHS general practice in England by headcount and FTE, with and without GP trainees. (b) Total GPs in NHS general practice in England per 1000 NHS patients by headcount and full-time-equivalent, with and without GP trainees. FTE = full-time equivalent. HC = headcount.

Between 2015 and 2024, using NHSE’s ‘All GPs’ category, which includes GP trainees, there was an 18% rise from 41 193 to 48 758 GPs (191/quarter [95% CI = 170 to 212]). Including GP trainees also produced peaks in September and troughs in June in the data related to when GP trainees start and end rotations in general practice. In contrast, counting fully qualified GPs alone produced a less steep rise of 6% from 36 082 to 38 124 (32/quarter [95% CI = 19 to 46]) with less fluctuation (Figure 1a, Supplementary Table 2).

### Total GPs by full-time equivalent, with and without GP trainees

Once reported working hours were considered the total number of FTE GPs, including GP trainees, only rose by 12% from 34 392 to 38 420 (96/quarter [95% CI = 73 to 122]). Excluding GP trainees, fully qualified FTE GPs fell by 5% from 29 364 to 27 966, an average loss of 62 FTE GPs per quarter (95% CI = −71 to −53) (Figure 1a, Supplementary Table 2).

### Total GPs per 1000 NHS patients, by headcount, with and without GP trainees

Between September 2015 and 2024, there was a 12% rise in the number of patients registered with an NHS general practice in England from 56 902 441 to 63 569 778 (174 573/quarter [95% CI = 169 422 to 179 722]). Therefore, once taking population growth into account, the rise in the number of GPs per capita, including GP trainees, was 6%, from 0.724 to 0.767 GPs/1000 patients (0.001/quarter [95% CI = 0.0007 to 0.0014]). Excluding GP trainees, there was a 5% reduction from 0.634 to 0.600 in fully qualified GPs/1000 patients (−0.0012 [95% CI = −0.0015 to −0.00099]) (Figure 1b, Supplementary Table S3).

### Total GPs per 1000 NHS patients, by full-time equivalent, with and without GP trainees

Considering reported working hours and including GP trainees, there was no overall rise in FTE GPs per capita. After removing GP trainees, a 15% reduction in fully qualified FTE GPs per capita was seen from 0.516 to 0.440 fully qualified FTE GPs/1000 patients (−0.0024/quarter [95% CI = −0.0025 to −0.0022]) (Figure 1b, Supplementary Table 3).

Including GP trainees and using headcounts produced a value 40% higher than if only counting fully qualified GPs and using FTE values in September 2015 (0.724 versus 0.516). By September 2024, this difference had increased to 74% (0.767 versus 0.440) (Table 1). This was a result of the number of doctors in GP training grades in NHS general practice more than doubling from 5142 to 10 823 (164/quarter [95% CI = 144 to 184]) between 2015 and 2024, and reported FTE hours falling, with the overall FTE to headcount ratio falling from 0.83 to 0.79, including GP trainees, and from 0.81 to 0.73, excluding GP trainees, over the 9-year period.

**Table 1.** Comparison of GPs in NHS general practice per 1000 NHS patients by FTE without GP trainees and by headcount with GP trainees between September 2015 and 2024

Definition of a GP per capita	September 2015	September 2024	Change over time, %
GPs excluding GP trainees FTE per 1000 patient	0.516	0.440	−15
GPs including GP trainees HC per 1000 patients	0.724	0.767	+6
Difference between FTE GPs without GP trainees and GPs by HC with GP trainees per 1000 patients, %	+40	+74	

HC = headcount; FTE = full-time equivalent;

### Comparison using ONS mid-year population estimates

In contrast to the number of patients registered with an NHS general practice, ONS mid-year population estimates were lower and increased at a slower rate, with a difference of 2.8 million (5%) in 2017 and 4.9 million (8%) by 2023 (Supplementary Figure S1). As a result, GP-to-patient ratios were higher and decreased at a slower rate using ONS population estimates, with there being 0.43 FTE fully qualified GPs in NHS general practice per 1000 NHS-registered patients versus 0.47 using ONS estimated population mid-2023 — in other words a difference of around 200 patients per GP (Supplementary Figure S2a and S2b).

### Range of NHS-registered patient-to-GP ratios across the country

**Figure 2.**
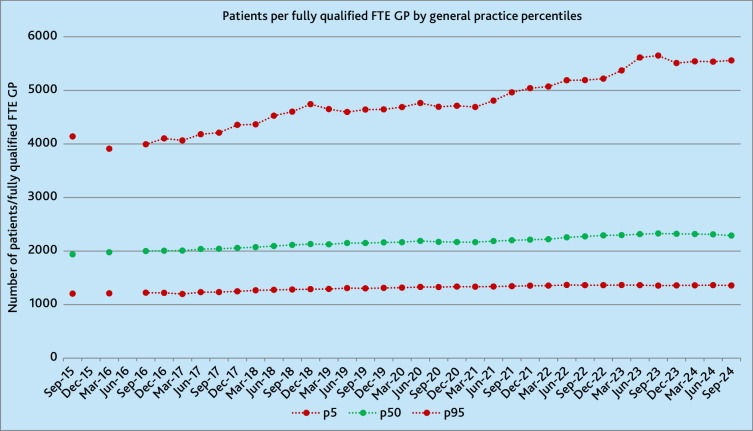
NHS-registered patients per fully qualified FTE GP, by NHS general practice percentiles. FTE = full-time equivalent. p5 = 5th percentile. p50 = 50th percentile. p95 = 95th percentile.

Over the 9-year period, the median number of NHS-registered patients per fully qualified FTE GP in NHS general practice rose 18% from 1938 to 2288 (10.5/quarter [95% CI 9.9 to 11.2]). In September 2015, the 5th and 95th percentiles of practices, respectively, had 1204 and 4139 patients per fully qualified FTE GP, whereas, in September 2024, the range had increased to 1357 and 5559. This widening difference was driven by the patient-to-GP ratio in the 95th percentile rising at a faster rate than that in the 5th percentile and resulted in the range between the 5th and 95th percentiles widening from 2936 to 4202 patients per fully qualified FTE GP, a 43% rise (Figure 2). Supplementary Figure S3 presents the same analysis by fully qualified FTE GPs per 1000 patients.

**Figure 3.**
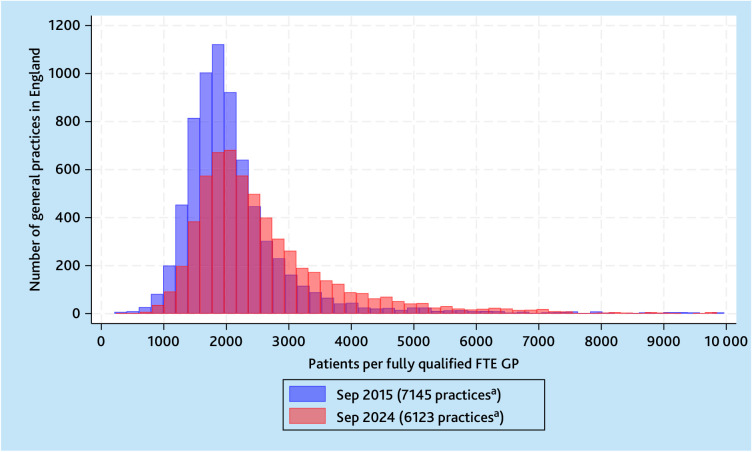
Comparison of histograms of the number of practices in September 2015 and September 2024 by NHS-registered patients per fully qualified FTE GP in NHS general practice. ^a^Seventy practices in 2015 and 81 practices in 2024 with >10 000 patients per fully qualified FTE GP have been excluded to visualise the histograms. FTE = full-time equivalent.

Figure 3 illustrates the right shift in the distribution of patients per fully qualified FTE GPs across general practices in England, with more practices having higher patient-to-GP ratios in September 2024 compared with September 2015.

## Discussion

### Summary

Between September 2015 and September 2024, subject to how a GP was defined and whether reported working hours were considered, the trend in the total number of GPs varied between an 18% rise or 5% reduction. Once changes in the number of patients registered in NHS general practice were considered, the trend in GPs per capita using NHS-registered patients varied between a 6% rise or 15% reduction. There was a substantial and increasing difference in the number of NHS-registered patients per GP between practices, with a 5th to 95th percentile range of 1204 and 4139 patients per fully qualified FTE GP in 2015 that increased to between 1357 and 5559 by 2024.

### Strengths and limitations

This is the first study, to the authors’ knowledge, that examines NHS general practice workforce data in England over time to understand how the picture of GP availability varies by method of calculation. There are, however, limitations to NHSE’s data. First, data submissions are dependent on practices logging in to the online NWRS platform to update information, and NHSE estimates data for practices that have not provided fully valid staff records — on average, 1.1% of GPs by headcount and 2.2% by FTE (including GP trainees) were estimated by NHSE owing to missing data between 2015 and 2024, and estimation rates were higher in 2015 and 2016.^3^ Also, although workforce submissions are mandatory and NHSE publishes the last time individual practices logged into the NWRS platform, NHSE does not report how many practices’ records in total across England may not be up to date.^8,10^

Second, reporting overtime hours — commonplace in general practice — would require continuous data capture.^11–15^ This is unlikely to be feasible or welcomed by practices, particularly for employed GPs if it would amount to an admission by employers of unpaid work. This is increasingly relevant as the proportion of salaried GPs in NHS general practice has risen since 2015, with salaried GPs representing 47% of fully qualified GPs by headcount and 40% by FTE in September 2024.^3,16^

Third, the total number of patients registered with an NHS general practice was higher than ONS census-based estimates of the size of the population in England and the difference increased over time. This generates uncertainty about which population count to use for workforce planning, although funding allocations to general practice are based on NHS-registered patients. NHS general practice list inflation is attributed to delayed deregistrations and duplicate registrations, but it is also recognised that undercoverage also exists because of, for example, un-registered migrants and existing patients being inappropriately removed under the ‘no-contact’ criteria.^17^ There may also be patients who are not registered with NHS general practice as they only use private health services. However, this number is likely to be small given the limited voluntary health insurance market in the UK and usual requirements for an NHS GP referral before accessing private specialist care.^18^

### Comparison with existing literature

There is widespread agreement that there is a shortage of GPs in NHS general practice and there have been successive government promises to increase numbers.^5,19–22^ However, the need to account for population growth, consider working hours, and the nuances of fully qualified GPs versus GP trainees are not consistently taken into account when reporting GP statistics.^4–6,23–25^ Some analyses also exclude regular locums on the basis that they are not ‘permanent’ GPs.^16,20,26,27^ Analysis of the cross-sectional National GP Worklife Surveys highlighted that in 2021 GPs reported working around 50% more time per contracted ‘session’. This increases the likelihood that reported NHS FTE hours are significantly underestimated as GPs’ workplans are usually defined by the number of ‘sessions’ they work and these are likely to be used by practice managers to populate FTE hours’ NWRS submissions.^12^ Differences between patients registered in NHS general practice and ONS population estimates were described 20 years ago; however, the widening discrepancy is concerning as it has implications for workforce planning and general practice funding, particularly in areas where discrepancies may be larger such as where there is greater list turnover.^28,29^

### Implications for research and practice

When citing NHS general practice GP statistics both GP headcount and FTE should be used. GP headcount alone will overestimate capacity, particularly as reported FTE hours are falling over time.^3,23^ A distinction should be made between fully qualified GPs and GP figures that include GP trainees. Including GP trainees overestimates current and future capacity, as GP trainees’ activities are not equivalent to those of fully qualified GPs and require fully qualified GP supervision time; NHSE’s GP trainee (‘GPs in Training Grades’) category includes foundation year doctors rotating through general practice, who may not choose to specialise in general practice; and based on current trends it is likely that a substantial proportion of GP registrars will not join the GP workforce full-time once qualified, if at all.^3,30–33^

Although regular locums are not in permanent employment, they represented 4% by headcount and 2% by FTE of the regular fully qualified GP workforce in NHS general practice in September 2024.^3^ Including regular locums in figures reflects fully qualified GP capacity and aligns with recent analysis of the same data by the ONS and other researchers.^23,25,34^ GP statistics using patients-to-GP ratios are useful to reflect capacity in the context of population growth, or decline, and reporting the range, such as between the 5th and 95th percentile of practices, is necessary to capture variation between practices and changes in this over time. The number of patients registered in NHS general practice is reported monthly at practice level and is used to calculate payments to general practice. It therefore seems more relevant to use this figure to calculate GP-to-patient figures than mid-year ONS estimates while NHSE should seek to address the discrepancies between the two sources.

Current FTE figures provide no insight into what proportion of GPs’ time is spent on direct clinical work (such as, appointments, clinical correspondence), indirect clinical work (such as, clinical meetings, audits, clinical supervision), practice management-related work (such as, staff employment, estates, finance), or operational problems (such as, IT glitches). Evidence from ethnographic case studies exists, but understanding these patterns across the country may offer greater transparency around GPs’ workload.^14,35,36^ Therefore, the collection of NHS general practice FTE GP hours could be improved by inviting individual GPs to cross-check and approve data on working hours submitted monthly by practices on their behalf, as well as indicate the nature of their work. The new NHS general practice GP appointments datasets could be cross-referenced with reported FTE GPs at practice level to better understand what proportion of GP time is spent on direct clinical care.^37^ NHSE could report on likely margins of error in GP numbers owing to out-of-date practice records on the NWRS portal. In addition, *ad hoc* locums figures (1865 by headcount in no other general practice role and 481 by FTE in September 2024^38^) that are currently provided in General Practice Workforce annex tables because of delays in the availability of these data should be brought into the main datasets, as well as the recently introduced PCN-employed GP roles, which although now reported in a combined General Practice and PCN Workforce experimental dataset called ‘Primary Care Workforce Quarterly’, remain separate from the principal General Practice Workforce datasets.^39,40^

It is very difficult to define a minimum acceptable workforce level for NHS general practices as there is considerable variation in practices’ skill mixes and population needs.^15,41^ However, being able to account for population need using indicators such as deprivation, age, gender, and multimorbidity whenever describing the workforce in general practice would allow policymakers, regulators, commissioners, and providers to better understand both overall trends in workforce capacity and inequities in distribution. In turn, this would help inform judgements about where quality of care and patient safety may be at higher risk because of GP shortages. Currently the Carr-Hill formula is used to weight practice populations for practice capitation payments. However, this has been criticised for not taking socioeconomic deprivation into sufficient account.^42–44^ Research to understand the most appropriate mechanism to account for a population’s general practice workforce needs would be helpful.

This analysis has focused on overall GP numbers in NHS general practice. Trends in NHS general practice GPs by role, gender, age band, and place of primary medical qualification, and comparison with the total number of General Medical Council-registered GPs in England, are published elsewhere.^24,45^ Likewise, examining the reasons for falling GP numbers and widening variation in NHS general practice were beyond the scope of the paper but other research has examined this.^46–53^ When counting and reporting on the rest of the general practice workforce, which is expanding compared with GPs, similar issues need to be considered. Correctly doing so may also help explain some of the variation in GP provision and would enable further research into how the balance of different roles influences quality, equity, and costs.^51,53^

In conclusion, there are numerous ways to report NHS general practice GP workforce statistics. This can result in contradictory discussions about trends and current figures. Reporting headcounts, including trainees in general practice, and ignoring population growth overestimates GP capacity and harms the interpretation of workforce trends. Using fully qualified FTE GPs per capita captures the current downwards trend in GP capacity, although there are limitations to current NHS data. Reporting the extent of variation across practices in England is necessary to capture the widening differences in GP provision.

## Data

All data are publicly available.
